# Sensitivity of metastatic mucinous tailgut cyst adenocarcinoma to gemcitabine and radiation: a case report

**DOI:** 10.3389/fonc.2025.1621324

**Published:** 2025-12-18

**Authors:** Maliha Nusrat, Kenneth H. Yu, Laura Tang, Mohammad Ali El Amine, Brian McCarthy, Karuna Ganesh, Elisa de Stanchina, Emmanouil Pappou, Julio Garcia-Aguilar, Meredith Bartelstein, Christopher Crane, David Kelsen, Leonard Saltz, Carla Hajj

**Affiliations:** 1 Gastrointestinal Oncology Service, Department of Medicine, Memorial Sloan Kettering Cancer Center, New York, NY, United States; 2 Department of Pathology and Laboratory Medicine, Memorial Sloan Kettering Cancer Center, New York, NY, United States; 3 Department of Radiology, Memorial Sloan Kettering Cancer Center, New York, NY, United States; 4 Adera Biolabs, Germantown, MD, United States; 5 The Antitumor Assessment Core Facility, Memorial Sloan Kettering Cancer Center, New York, NY, United States; 6 Department of Surgery, Memorial Sloan Kettering Cancer Center, New York, NY, United States; 7 Department of Radiation Oncology, Memorial Sloan Kettering Cancer Center, New York, NY, United States

**Keywords:** tailgut cyst adenocarcinoma, retrorectal cyst hamartoma, circulating tumor cells, chemosensitivity assay, personalized medicine

## Abstract

Metastatic mucinous adenocarcinoma arising from a tailgut cyst is an ultra-rare cancer. Literature on the chemosensitivity and radiosensitivity of this cancer is lacking, and treatment is extrapolated from rectal cancer management. We are reporting, for the first time, the case of a patient with metastatic tailgut cyst adenocarcinoma who derived 1.5 years of clinical benefit from treatment with gemcitabine, selected as a result of transcriptomic analysis of her circulating tumor cells using an *in vitro* assay. The cancer was refractory to regimens extrapolated from rectal cancer management (capecitabine with oxaliplatin, and irinotecan). Pelvic recurrence and osseous metastases were clearly radiosensitive. Tumor molecular profile showed microsatellite stable cancer with *KRAS* p.G13D and *TP53* p.C176W mutations.

## Introduction

Tailgut cyst or retrorectal cyst hamartoma is a congenital remnant of the embryonic tailgut (1). The developing human embryo has a tail caudal to the cloaca (future anorectal canal), and a portion of the hindgut extends into this tail as the tailgut or post-anal gut (**Supplementary Figure S1**) (2). Failure of the tailgut to involute by the eighth week of gestation can result in cysts. True incidence is under-estimated, as many tailgut cysts are asymptomatic, and it is at least one in 40,000 hospital admissions (3). Patients may present with constipation, perineal pain, blood in stool, perianal fistulas or abscesses, and dysuria. Tailgut cysts are more frequently seen in women, with female-to-male ratios ranging from 3:1 to 9:1 (1, 4, 5).

Malignant transformation of tailgut cysts can occur in 6%–26% of cases (4–7). Varied cancer histologies include mucinous adenocarcinoma, squamous cell, neuroendocrine, transitional cell, endometroid adenocarcinoma, sarcoma, and mixed (such as adenosquamous carcinoma) (7, 8). This histologic diversity is reflective of how the distal hindgut develops (**Supplementary Figure S1**). By week 4 of gestation, the human embryo develops a gut tube divided into the foregut, midgut, and hindgut (9). Cloaca is the expanded distal portion of the hindgut, which terminates at the cloacal membrane at the junction of the endoderm and ectoderm (proctodeum). The proctodeum develops stratified squamous epithelium of the outer one-third of the anal canal. The urorectal septum grows into the cloaca and divides it into the urogenital sinus anteriorly and the anal canal posteriorly. Neural crest cells migrate into the bowel wall, completing migration into the hindgut in the seventh week of gestation.

Metastatic mucinous adenocarcinoma arising from tailgut cyst (mMATC) is ultra-rare and has been reported with poorly differentiated histology, positive margin resection, and intraoperative cyst leakage (10–15). The reported sites of metastasis of tailgut cyst adenocarcinoma include the lungs, peritoneum, bone, liver, and inguinal lymph nodes (10–15). Literature on the chemosensitivity and radiosensitivity of mMATC is lacking; treatment is extrapolated from rectal cancer management. We are reporting, for the first time, 1.5 years of clinical benefit from gemcitabine, and providing clear evidence of radiosensitivity.

## Case presentation

**Figure 1:**
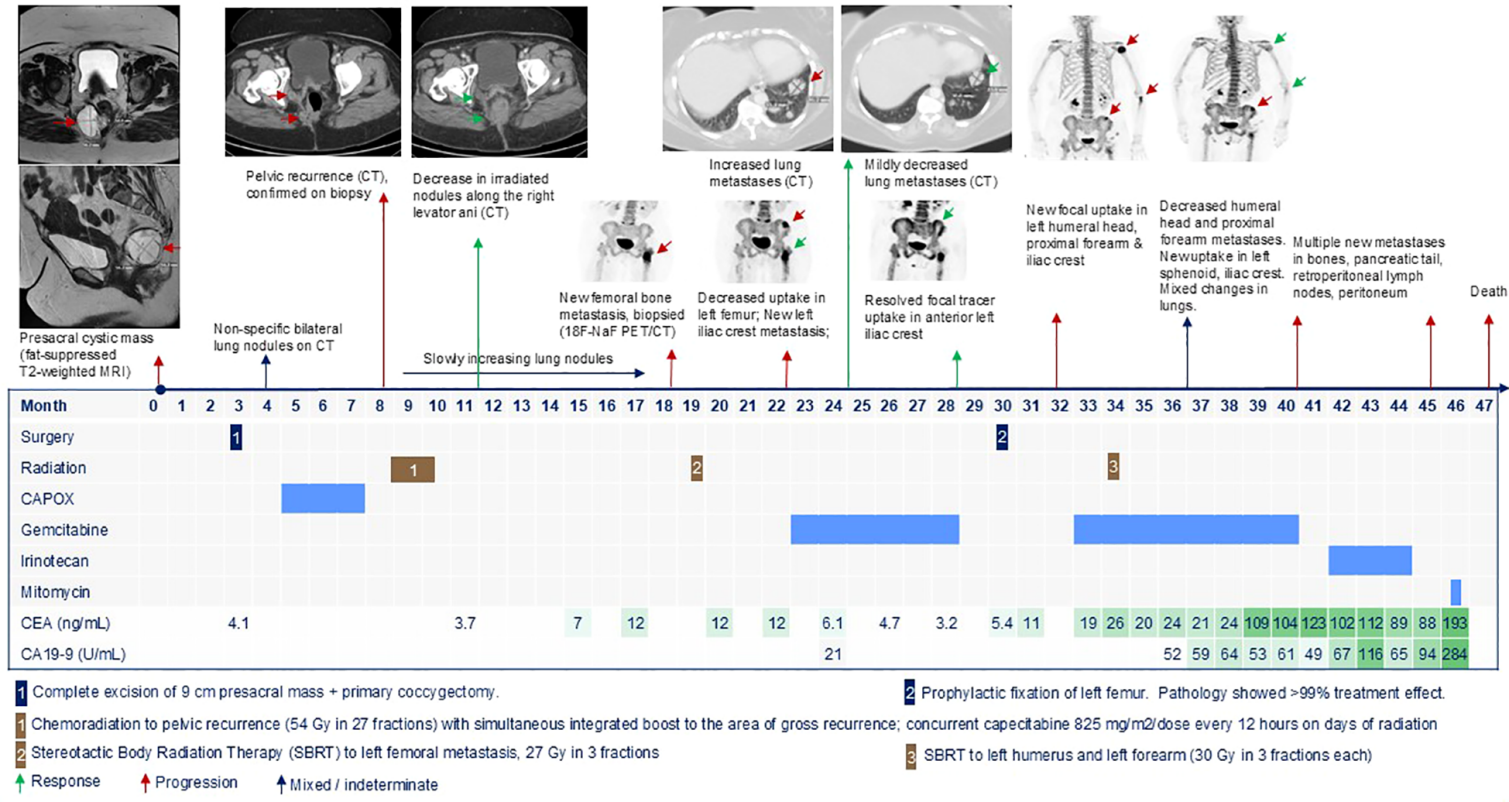
Timeline of patient’s oncologic history.

**Figure 2:**
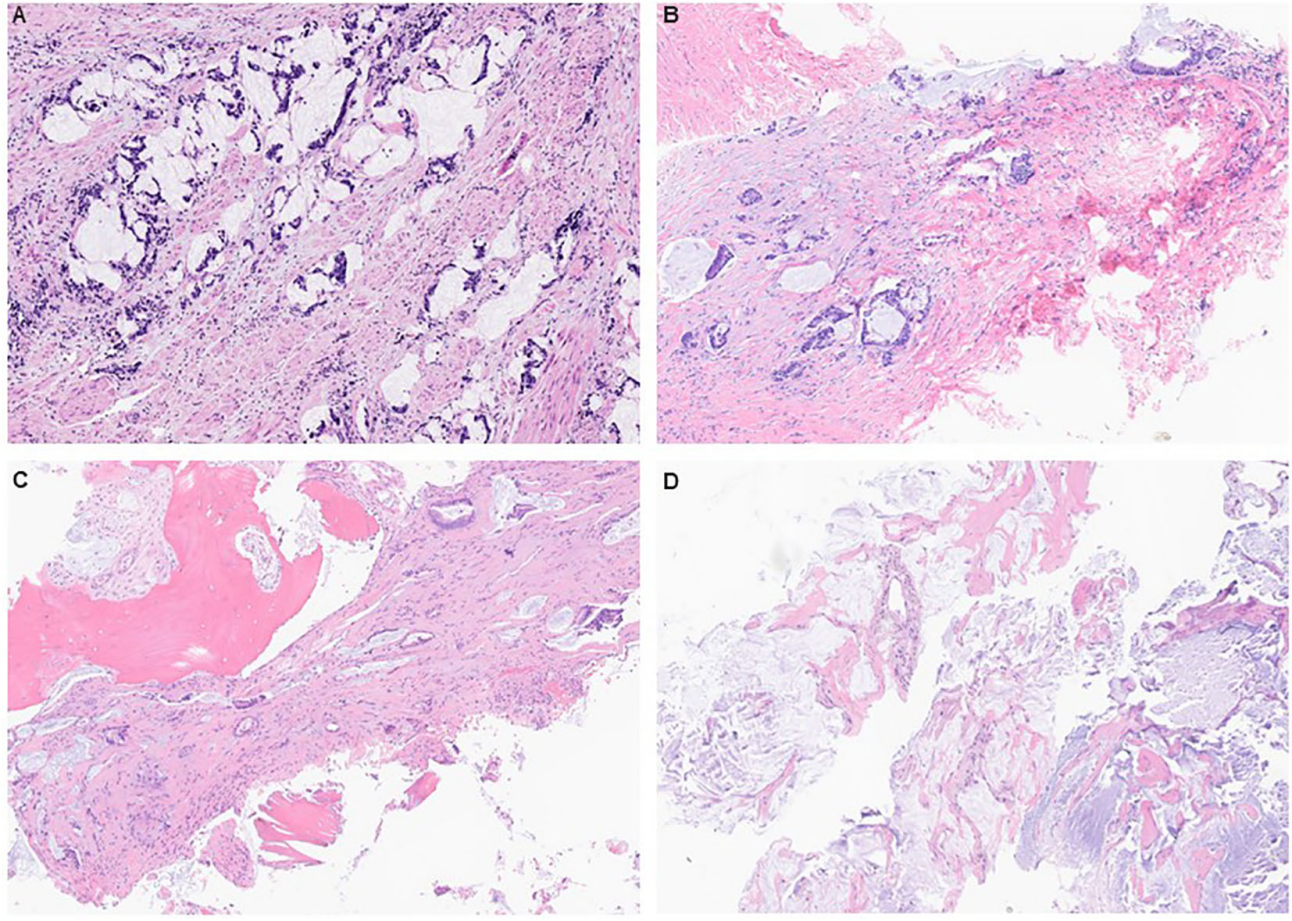
Histopathology of primary tumor and metastatic specimens showed moderate to poorly differentiated mucinous adenocarcinoma arising in tailgut cyst. **(A)** Surgical excision of the presacral mass with primary coccygectomy. **(B)** Biopsy of recurrent pelvic nodules. **(C)** Biopsy of left femoral mass. **(D)** Biopsy of left lung mass showing acellular mucin pools similar to the prior presacral mass specimen.

A 57-year-old woman, with obesity (body mass index 40.4 kg/m^2^) and a history of perianal abscess 4 years prior, presented with a 1-year history of constipation and intermittent perineal pain aggravated by prolonged sitting. Magnetic resonance imaging (MRI) showed a lobulated, multiseptated presacral cystic mass measuring 5.8 × 5.7 × 5.6 cm with mildly nodular enhancement of internal septations, inseparable from the wall of the lower rectum, probable tailgut duplication cyst (**Figure 1**). Flexible sigmoidoscopy showed extraluminal compression of the posterior midline rectum approximately 5 cm from the anal verge. She underwent surgical excision of the presacral mass with primary coccygectomy. The patient was placed in a padded prone jack-knife position, and a midline presacral incision was made from the tip of the coccyx to just above the anal sphincter complex. Intraoperatively, a large 9-cm presacral mass was found, densely adherent to the rectum and coccyx. The mass was carefully dissected from the rectum, vagina, gluteal muscle, and surrounding subcuticular fat and was removed completely. Pathology confirmed moderate to poorly differentiated adenocarcinoma with mucinous differentiation and perineural invasion (**Figure 2A**). The area with the suture was also positive for adenocarcinoma. Remnants of a tailgut cyst were present. Immunohistochemistry was positive for CK20, CDX2, and CK7. Progesterone receptor highlighted smooth muscle and rare stromal cells.

### Resistance to CAPOX

Post-operative computed tomography (CT) scan showed a new right common iliac node 2.6 × 2.1 cm and a few bilateral non-specific pulmonary nodules (largest 1.1 cm). The case was discussed at the colorectal disease management team meeting with consensus to pursue adjuvant chemotherapy with capecitabine 1,000 mg/m^2^/dose oral every 12 hours on days 1–14 + oxaliplatin 130 mg/m^2^ intravenous (IV) on day 1 of every 21 days (CAPOX) for four cycles, followed by concurrent chemoradiation treatment to the surgical bed and lymph nodes, as an extrapolation from rectal cancer management. After completion of four cycles of CAPOX, the CT scan showed new nodules in the surgical bed and a slight increase in the size of lung nodules suspicious for metastases (stage IV). A biopsy of the right perirectal soft tissue showed moderately differentiated adenocarcinoma with mucinous features, as seen in the presacral mass (**Figure 2B**).

### Response to radiation

The patient underwent chemoradiation treatment to the pelvis with a simultaneous integrated boost to the area of gross disease recurrence (54 Gy in 27 fractions). She received concurrent capecitabine 825 mg/m^2^/dose every 12 hours on radiation days. Radiotherapy was well-tolerated, and the irradiated pelvic nodules decreased on subsequent imaging. Slowly growing asymptomatic lung metastases were observed.

Six months after chemoradiation treatment, she developed left leg pain and an increase in Carcinoembryonic Antigen (CEA) level. A subsequent MRI showed probable left femoral sclerotic metastasis. ^18^F-Sodium fluoride positron emission tomography–CT (^18^F-NaF PET/CT) showed no other bone lesions. CT-guided core needle biopsy of the left femoral lesion confirmed metastatic mucinous adenocarcinoma, morphologically similar to the prior material (**Figure 2C**). She underwent stereotactic radiation to the left femoral metastasis (27 Gy in 3 fractions). Three months later, ^18^F-NaF PET/CT showed decreased uptake in the sclerotic metastasis of the proximal left femur, but a new tracer-avid lytic metastasis in the left anterior iliac crest. The CT scan showed continued slow growth of lung metastases. Given that there is no literature on the chemosensitivity of this cancer, the patient deferred systemic therapy and obtained consultations at other cancer centers. No suitable therapeutic clinical trial was available.

### Molecular studies

Next-generation sequencing of the primary tumor was performed on the MSK-IMPACT panel to detect single-nucleotide variants and small insertions and deletions (<30 bp) in protein-coding exons of the 505 genes, as previously described (16). The results showed microsatellite-stable cancer with tumor mutation burden of 1.6 mutations/megabases, *KRAS* p.G13D, *TP53* p.C176W. No copy number alterations or structural variants were seen. Germline DNA analysis of 90 genes on MSK-IMPACT showed a heterozygous pathogenic variant in *MUTYH* p.G396D (monoallelic mutation carrier). No gene fusions were found on MSK-Archer FusionPlex™ Custom Solid Panel of 123 genes. Generation of patient-derived organoids and xenografts was attempted from the biopsy of the pelvic recurrence, but the preclinical models were not established.

**Figure 3:**
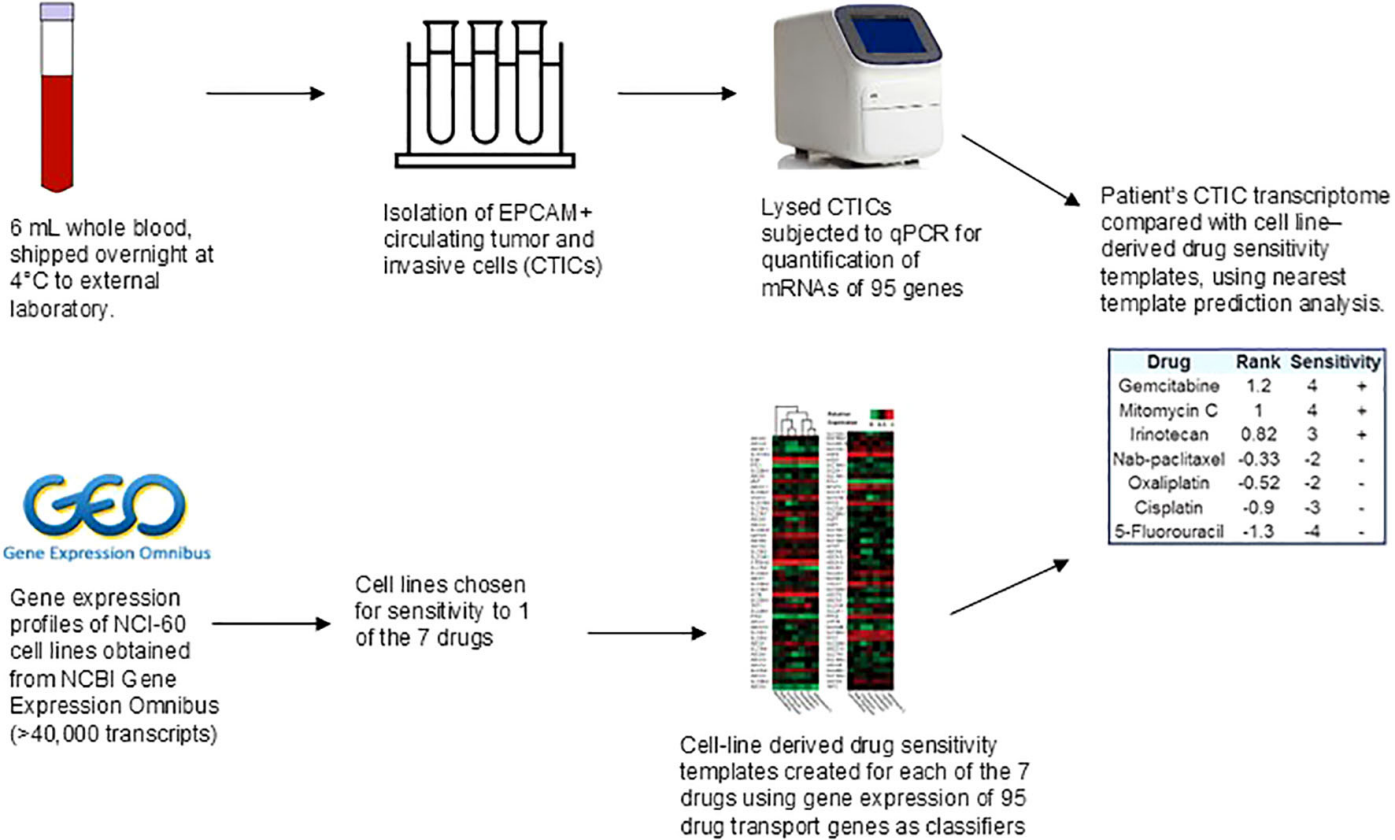
Transcriptomic analysis of circulating tumor and invasive cells to determine chemosensitivity or resistance. Drug sensitivity templates were created for seven chemotherapeutic agents (5-fluorouracil, irinotecan, oxaliplatin, cisplatin, gemcitabine, nab-paclitaxel, and mitomycin C) using gene expression patterns of NCI-60 cell lines. Cell lines were selected for sensitivity to one of these seven chemotherapy drugs. Gene expression profiles of 95 drug transport genes were used as classifiers in the algorithm to generate drug sensitivity templates. The chemotherapy predictive value of the assay was previously validated in pancreatic adenocarcinoma (17, 18). Circulating tumor and invasive cells (CTICs) were isolated from patient’s whole blood, lysed, and subjected to quantitative polymerase chain reaction (qPCR) to determine messenger RNA (mRNA) levels for 95 drug transport genes. The patient’s CTIC transcriptome was compared with cell line-derived drug sensitivity templates using nearest template prediction analysis. The underlying premise of the nearest template prediction analysis is that if two samples share similar expression profiles of relevant genes, then their response to drugs will be similar. The templates were sorted in rank order to obtain sensitivity values of each of the seven chemotherapy drugs, as previously described (17, 18).

Six milliliters of the patient’s whole blood was used for the transcriptomic profiling of circulating tumor and invasive cells (CTICs) to investigate chemosensitivity to seven drugs used in the management of gastrointestinal and genitourinary cancers, as previously described (**Figure 3**) (17, 18). The patient’s heparinized whole blood was shipped at 4°C overnight to Adera Biolabs (Germantown, MD, USA). A collagen adhesion matrix in a modified cell invasion assay was used to capture EPCAM+ invasive cells; 1.5 mL aliquots of whole blood were incubated with collagen-coated microcarriers (Pall Corporation, Port Washington, NY, USA) and cultured for 2 hours in Dulbecco’s modified Eagle’s medium with F12 supplemented with 10% calf serum, 5% Nu-serum, 1 unit/mL penicillin, and 10 μg/mL streptomycin. Captured cells were then washed and lysed *in situ*. Total RNA from lysed cells was purified using the RNeasy Mini Kit (Qiagen, Valencia, CA, USA). cDNA was synthesized (Ovation Pico SL, Nugen Technologies, San Carlos, CA, USA) and then subjected to quantitative polymerase chain reaction to measure messenger RNA levels of 95 drug transport genes at standard thermal cycling rates using standard SYBR Green and ROX Mastermix. Arrays with >5% error rates were discarded. Assay reliability was assessed in accordance with Clinical Laboratory Improvement Amendments (CLIA)-certified mandates. Drug sensitivity templates were previously created for seven chemotherapeutic agents using publicly available gene expression data of NCI-60 cell lines. The patient’s CTIC transcriptome was compared with cell line-derived drug sensitivity templates using nearest template prediction analysis, as previously described (17, 18).

The results of the chemosensitivity assay, obtained in 5–10 days, showed sensitivity to gemcitabine, mitomycin C, and irinotecan, and resistance to 5-fluorouracil, oxaliplatin, cisplatin, and nab-paclitaxel. The concordance between the assay-predicted resistance to 5-fluorouracil and oxaliplatin and the patient’s clinical progression on CAPOX was notable. After a detailed discussion of the investigational nature of the chemosensitivity assay and the potential side effects of gemcitabine and irinotecan, the patient decided to proceed with gemcitabine due to the risk of alopecia with irinotecan.

### Response to gemcitabine and radiation

She received gemcitabine 1,000 mg/m^2^ IV on days 1 and 8 of every 21 days. She experienced flu-like symptoms and erythematous rash post-infusion, which were managed with low-dose dexamethasone. After 2 months, the bilateral lung metastases decreased in size. By 4 months, CEA normalized, and the anterior left iliac crest metastasis resolved on imaging.

Gemcitabine was held after 6 months for prophylactic fixation of the previously irradiated left femur due to leg pain. Pathology showed metastatic mucinous adenocarcinoma with >99% treatment effect. Two months after surgery, ^18^F-NaF PET/CT showed new focal increased uptake in the left humeral head, proximal forearm, and iliac crest. CEA increased. Painful left humerus and radial metastases responded well to stereotactic radiation.

After resuming gemcitabine, CEA stabilized. Over the next 6 months, lung metastases increased by 2–3 mm, and there was suspicion of new/increased bone metastases in the sphenoid and the left iliac crest. CT-guided biopsy of growing lung metastasis was performed to reattempt preclinical models, but showed acellular mucin pools similar to the prior presacral mass (**Figure 2D**).

### Gemcitabine-induced radiation recall

Subsequently, the patient experienced worsening left-sided arm and leg pain and edema. MRI of the left shoulder and forearm showed treated metastases; post-radiation myositis and myonecrosis in the clavicular and acromial portion of the deltoid, the elbow, and the forearm; elbow synovitis; and diffuse subcutaneous edema and skin thickening. MRI of the left femur showed probable proximal sciatic neuritis at the level of the prior radiation field and scar tissue causing nerve entrapment (**Supplementary Figure S2**). Myositis was attributed to gemcitabine-induced radiation recall. Symptoms improved with oral methylprednisolone and supportive care.

### Progression on irinotecan and mitomycin C

Eight months after resuming gemcitabine, imaging showed new osseous metastases, peritoneal nodules, and retroperitoneal lymphadenopathy. She received irinotecan 180 mg/m^2^ IV every 2 weeks and experienced diarrhea and fatigue requiring dose reduction to 155 mg/m^2^ after two doses. Although tumor markers downtrended, imaging after six doses of irinotecan showed progression in distant lymph nodes, peritoneum, pleura, bones, and pelvis. She received one dose of mitomycin 5 mg/m^2^ IV. Three weeks later, she was admitted with hoarseness and breathlessness due to cancer progression. She was transitioned to hospice and passed away.

## Discussion

This is the first detailed report of mMATC describing sensitivity to gemcitabine and excellent response to radiotherapy at all irradiated sites. We found only six cases of mMATC previously reported in the literature, and these are summarized in **Table 1**; treatment data are extremely limited. In our patient, the initial site of metastases was the lung, then bone, followed by the peritoneum, distant lymph nodes, pleura, and pancreas. Bone metastases were sclerotic and lytic. CEA and Carbohydrate Antigen 19-9 (CA 19-9) levels correlated with progression, as previously reported (10).

**Table 1:** Published cases of metastatic tailgut cyst adenocarcinoma.

Ref	Age (years)	Gender	Presentation	Initial management	Histology	Post-op time to recurrence	Metastatic site	CEA (ng/mL)	CA 19-9 (U/mL)	Treatment	Outcome
(10)	40	Woman	Severe perineal pain for 1 month	• Biopsy • En bloc resection of the mass with Hartmann operation + coccygectomy + partial sacrectomy to the level of S4 through a combined abdominal/sacral approach • Adjuvant RT	• Biopsy: Grade 3 carcinoma with focal squamous differentiation • Surgical: Grade 3 adenocarcinoma	5 months	Inguinal lymph node, local recurrence involving sacrum	↑ Pre-op; normal post-op; ↑ on recurrence	↑ Pre-op; normal post-op; ↑ recurrence	5-FU-based chemotherapy	Unknown
(11)	38	Man	Increased urinary and stool frequency and urgency, abdominal fullness, and pain for 6 months	• FNA • Surgical excision (not en bloc)	• FNA: adenocarcinoma • Surgical: Grade 3 adenocarcinoma	6 months	Lungs	n/a	n/a	n/a	Lost to follow-up
(12)	71	Woman	Tailbone/pelvic pain for few months	• Incomplete resection (80% removed) • Adjuvant 5-fluorouracil, Leucovorin, abd oxaliplatin (FOLFOX) → chemoRT with 5-FU infusion (total 6 months)	• Surgical: adenocarcinoma	13 months	Peritoneum	n/a	n/a	Hospice	Hospice
(13)	37	Woman	Abdominal discomfort, vaginal bleeding, and rectal fullness for 2 years	• Resection of left-sided pre-sacral mass + appendectomy + left oophorectomy (complete, but not en bloc) • Adjuvant FOLFOX + Avastin → capecitabine + carboplatin	• Surgical: Grade 1 mucinous adenocarcinoma. Appendix and ovary were not involved	16 months	Pseudomyxoma peritonei	n/a	n/a	Complete cytoreductive surgery (total abdominal hysterectomy, bilateral salpingectomy, right oophorectomy, greater omentectomy, lesser omentectomy, pelvic peritonectomy, splenectomy, and left ureterolysis) → hyperthermic bidirectional chemotherapy with intraperitoneal mitomycin C and doxorubicin + systemic 5-FU and leucovorin	Disease-free 19 months post-surgery
(14)	63	Woman	Progressive gluteal swelling and chronic lower back pain for few months	• FNA • Resection through a posterior approach	• FNA: no cancer • Surgical: mucinous adenocarcinoma. Margin positive	Immediate	Inguinal lymph node, local recurrence, bone	↑ After metastasis	↑ After metastasis	Post-op scan showed 1.4 cm inguinal lymph node CAPOX ×5 cycles → mild reduction in lymph node (size not mentioned) → local recurrence after 4 months → RT → stable pelvic recurrence → bone metastases → capecitabine → progression	Died 28 months after diagnosis
(15)	47	Man	Bilateral flank pain, strangury, obstipation, and changing stool calibers for 3 months	• Biopsy • Complete resection of the mass, with *in toto* coccygectomy + partial sacrectomy to the level of S3, through a combined laparotomy and posterior pelvic incision approach	• Biopsy: Grade 3 adenocarcinoma • Surgical: moderately well-differentiated adenocarcinoma	6 months	Liver, lungs	↑ Pre-op; normal post-op, ↑ on recurrence	n/a	Chemotherapy (details unknown)	Died 14 months after diagnosis

Risk factors for metastases are shown in red font. The cell highlighted green shows positive patient outcome.

5-FU, 5-fluorouracil; FNA, fine-needle aspiration; op, operative; RT, radiotherapy; CAPOX, capecitabine + oxaliplatin; CEA, carcinoembryonic antigen; CA 19-9, carbohydrate antigen 19-9; FOLFOX, 5-fluorouracil, Leucovorin, and oxaliplatin; Ref, reference.

Most tailgut cysts are multicystic, lined by a variety of epithelia, including squamous, columnar, transitional, and cuboidal, with scattered enterochromaffin cells (1). The embryonic cloaca is pluripotent (**Supplementary Figure S1**). Hence, chemosensitivity of mMATC may resemble that of genitourinary, gynecologic, colorectal, or anal cancer. This was reflected in our patients’ chemosensitivity assay results that showed sensitivity to gemcitabine, mitomycin, and irinotecan. Our patient obtained clinical benefit from gemcitabine, but did not benefit from the colorectal cancer regimens; further investigation is warranted to confirm these findings in other patients.

Optimizing growth conditions for preclinical models of ultra-rare cancers is challenging. Patient-derived xenografts and organoids did not establish successfully from our patient’s cancer. Novel *in vitro* assays for personalized chemotherapy selection in real-time have the potential to improve patient survival. The *in vitro* chemosensitivity assay used for our patient required 6 mL of whole blood and generated results in 5–10 days, which was convenient. The detailed methodology and earlier validation studies have been previously described (17, 18). This assay has shown predictive performance for time to progression on chemotherapy and overall survival in small prospective cohorts of patients with metastatic pancreatic cancer and is being further validated (17). Although the assay predicted sensitivity to irinotecan and mitomycin C, our patient did not benefit from these drugs. It is plausible that chemosensitivity changed after the first regimen, and initial results may not accurately predict outcome on subsequent lines of therapy.

This case also illustrates the radiosensitivity of mMATC. Prior literature is limited to adjuvant, neoadjuvant, or locally recurrent settings, without clear response data (10, 19–21). Our patient experienced a rare radiation recall myositis from gemcitabine, characterized by an inflammatory reaction within the irradiated fields during chemotherapy (22). Skin edema and limb pain resolved with initiation of steroids and discontinuation of gemcitabine, but restricted mobility of the left shoulder and hip persisted, requiring physical and occupational therapy.

Genomic analysis showed somatic *TP53* inactivating and *KRAS* activating mutations, implying oncogenesis through the mitogen-activated protein kinase signaling pathway. A germline heterozygous *MUTYH* p.G396D mutation was seen, which is a loss-of-function mutation annotated as likely oncogenic in the OncoKb database (23). Monoallelic *MUTYH* germline variants confer a 0.46% risk of colorectal cancer by age 45–49 years, comparable to the average risk (24). Somatic loss of heterozygosity in *MUTYH* heterozygotes may increase the risk of various cancers (25). However, the association between germline *MUTYH* variants and tailgut cyst carcinogenesis is unknown.

We acknowledge the limitations of this case report, which include the fact that clinical outcomes related to one case warrant further investigation before treatments can be generalized to other patients with this cancer, and this emphasizes the need for international registries for sharing preclinical knowledge and clinical experiences in ultra-rare cancers. We could not successfully generate preclinical models (patient-derived xenografts and organoids) to pursue in-depth investigation of the mechanisms behind the observed clinical outcomes, and this remains an area for future research. Lastly, the specific algorithm used to create drug sensitivity templates is proprietary to the laboratory that performed the assay and is not included in the manuscript. Nevertheless, this case report meaningfully contributes to the literature on the management of the ultra-rare metastatic mucinous tailgut cyst adenocarcinoma, providing a detailed clinical history and evidence of response to gemcitabine in this patient, which is usually not used in the management of mMATC, as treatment is extrapolated from colorectal cancer management. The case also provides evidence of the radiosensitivity of mMATC, implying the potential role of radiation in neoadjuvant, adjuvant, locally recurrent, and advanced disease settings. These clinical observations require confirmation in larger studies.

## Conclusions

mMATC is an ultra-rare cancer that showed sensitivity to gemcitabine and radiation in our patient. The benefit from other chemotherapies is unproven. Further investigation is warranted for the generalizability of outcomes to other patients with mMATC. International registries are needed for sharing preclinical knowledge and clinical experiences in ultra-rare cancers.

## Data Availability

The original contributions presented in the study are included in the article/**Supplementary Material**. Further inquiries can be directed to the corresponding author.
