# Supplementary material for: Sensitivity of metastatic mucinous tailgut cyst adenocarcinoma to gemcitabine and radiation: a case report
Source: Front Oncol. 2025 Dec 18;15:1621324. doi: 10.3389/fonc.2025.1621324 (PMC12756884; doi:10.3389/fonc.2025.1621324)
Supplement: Supplementary file 1 [file Presentation1.pptx]

## Slide 1
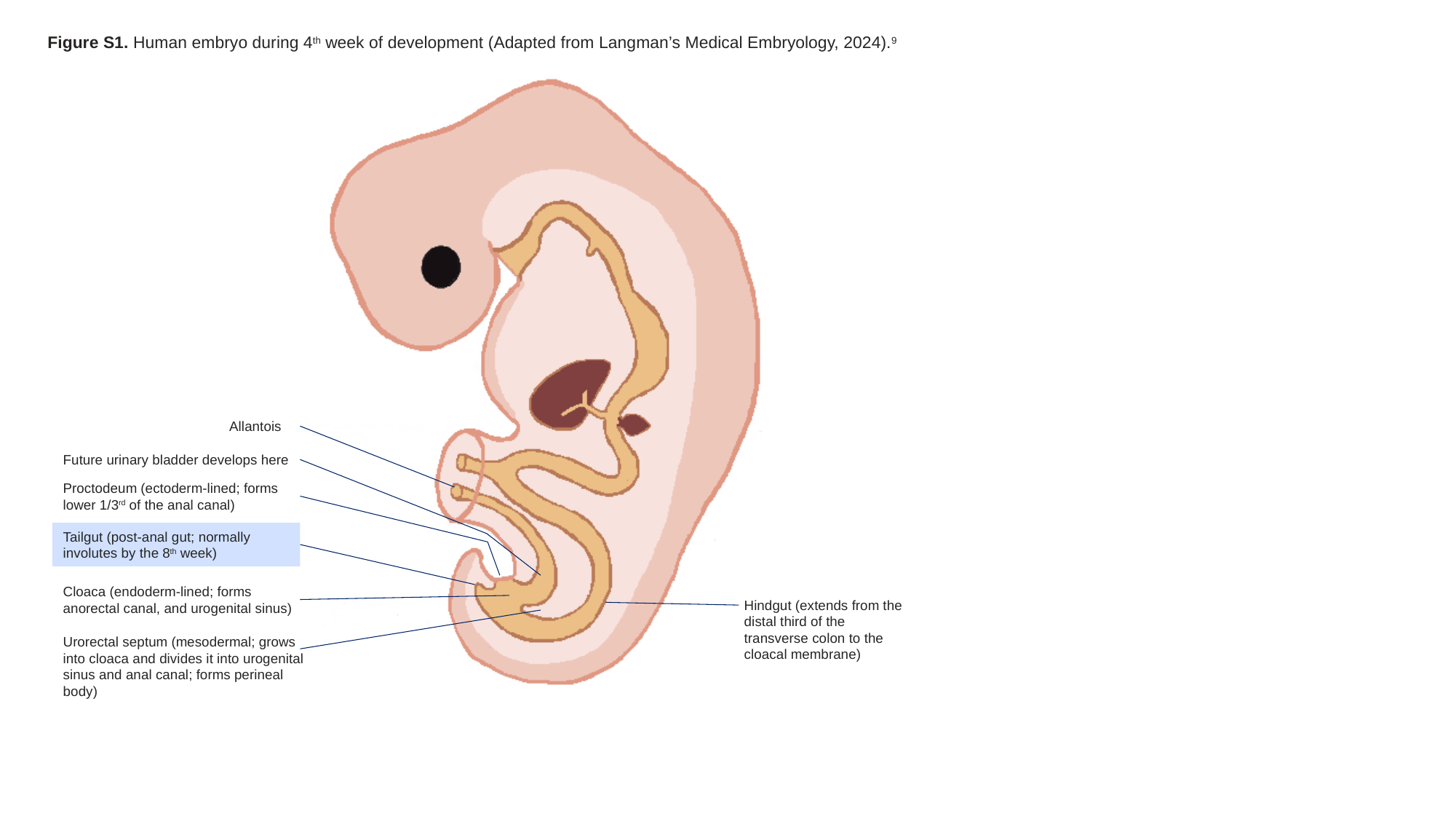

Figure S1. Human embryo during 4th week of development (Adapted from Langman’s Medical Embryology, 2024).9
Allantois
Future urinary bladder develops here
Proctodeum (ectoderm-lined; forms lower 1/3rd of the anal canal)
Tailgut (post-anal gut; normally involutes by the 8th week)
Cloaca (endoderm-lined; forms anorectal canal, and urogenital sinus)
Hindgut (extends from the distal third of the transverse colon to the cloacal membrane)
Urorectal septum (mesodermal; grows into cloaca and divides it into urogenital sinus and anal canal; forms perineal body)

## Slide 2
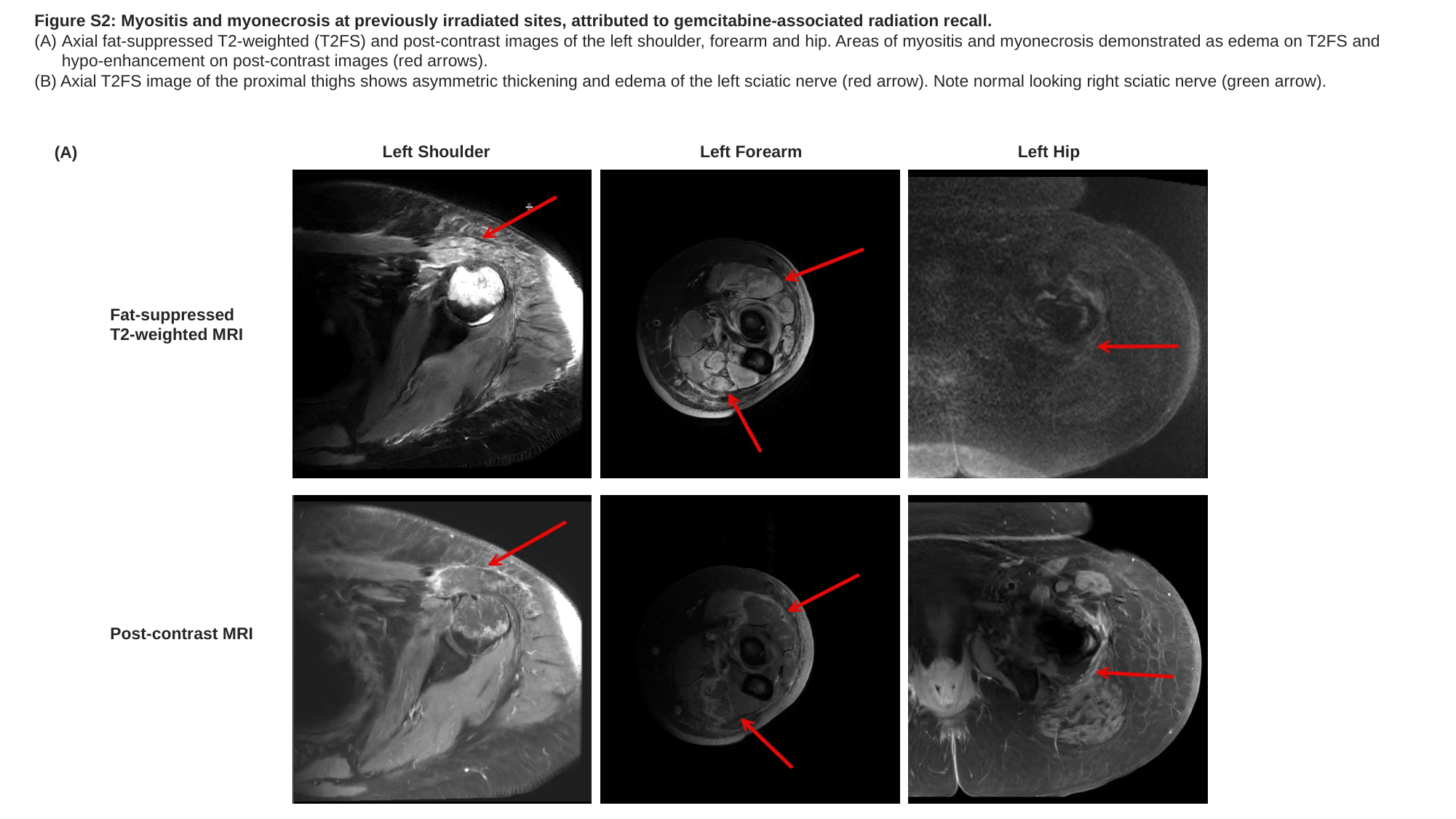

Figure S2: Myositis and myonecrosis at previously irradiated sites, attributed to gemcitabine-associated radiation recall.
Axial fat-suppressed T2-weighted (T2FS) and post-contrast images of the left shoulder, forearm and hip. Areas of myositis and myonecrosis demonstrated as edema on T2FS and hypo-enhancement on post-contrast images (red arrows).
(B) Axial T2FS image of the proximal thighs shows asymmetric thickening and edema of the left sciatic nerve (red arrow). Note normal looking right sciatic nerve (green arrow).
Left Shoulder
Left Forearm
Left Hip
(A)
Fat-suppressed
T2-weighted MRI
Post-contrast MRI

## Slide 3
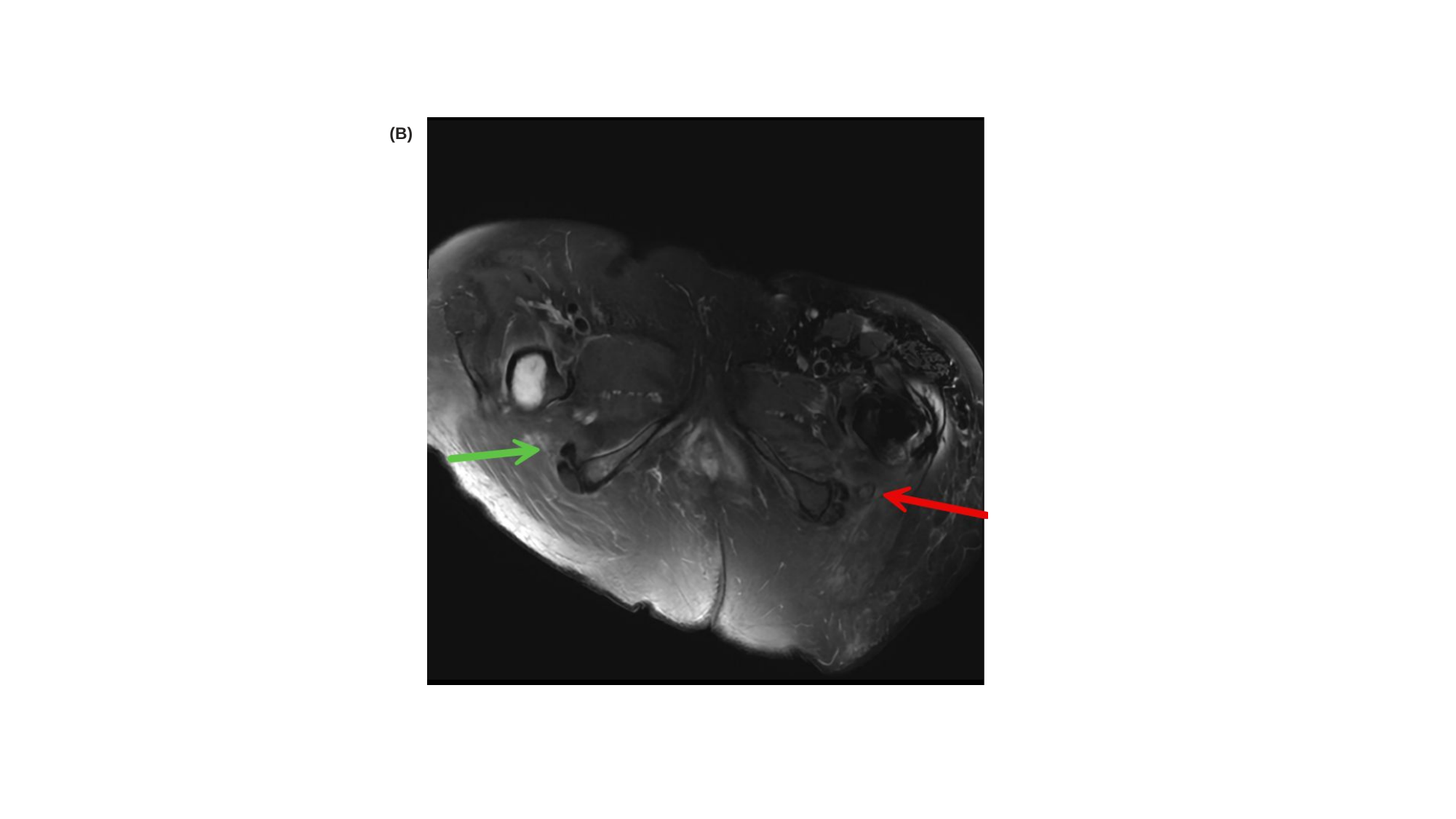

(B)
